# Sellar Glomus Tumor Misdiagnosed as Pituitary Adenoma: A Case Report and Review of the Literature

**DOI:** 10.3389/fendo.2022.895054

**Published:** 2022-05-04

**Authors:** Yijun Cheng, Hao Tang, Zhe Bao Wu

**Affiliations:** Department of Neurosurgery, Center of Pituitary Tumor, Ruijin Hospital, Shanghai Jiao Tong University School of Medicine, Shanghai, China

**Keywords:** sellar, glomus tumor, hypopituitarism, pituitary adenoma, endoscopic transsphenoidal approach

## Abstract

Glomus tumor is a rare mesenchymal tumor with an organ-like structure. Sellar glomus tumors are extremely rare with only six reported cases in the literature. Because of the lack of special clinical manifestations and imaging features, the disorder may be easily misdiagnosed as other sellar tumors, especially pituitary adenomas. Here, the present study showed a case of a 69-year-old male with hypopituitarism who was preliminarily misdiagnosed as non-functional pituitary adenoma.

## Introduction

Sellar tumors consist of a broad range of benign and malignant lesions due to the complex anatomy of the sellar region. Notably, many of the sellar tumors are newly described or have recently revised nomenclature in the 2017 Revision of the World Health Organization (WHO) classification system (1). Despite this wide range, approximately 80% of sellar tumors are due to the five most common lesions, including the pituitary adenomas, meningiomas, aneurysms, glioma, and craniopharyngiomas (2). In particular, pituitary adenomas are the most common sellar tumors that can account for as high as 10%–15% of all intracranial tumors. Compared to other intracranial lesions, imaging features for sellar tumors are relatively less specific, which always results in a misdiagnosis (3). Here, we report an extremely rare case of sellar glomus tumors in a 69-year-old male who was misdiagnosed as non-functional pituitary adenoma with hyperthyroidism for more than 5 years.

## Clinical Presentation

**Figure 1:**
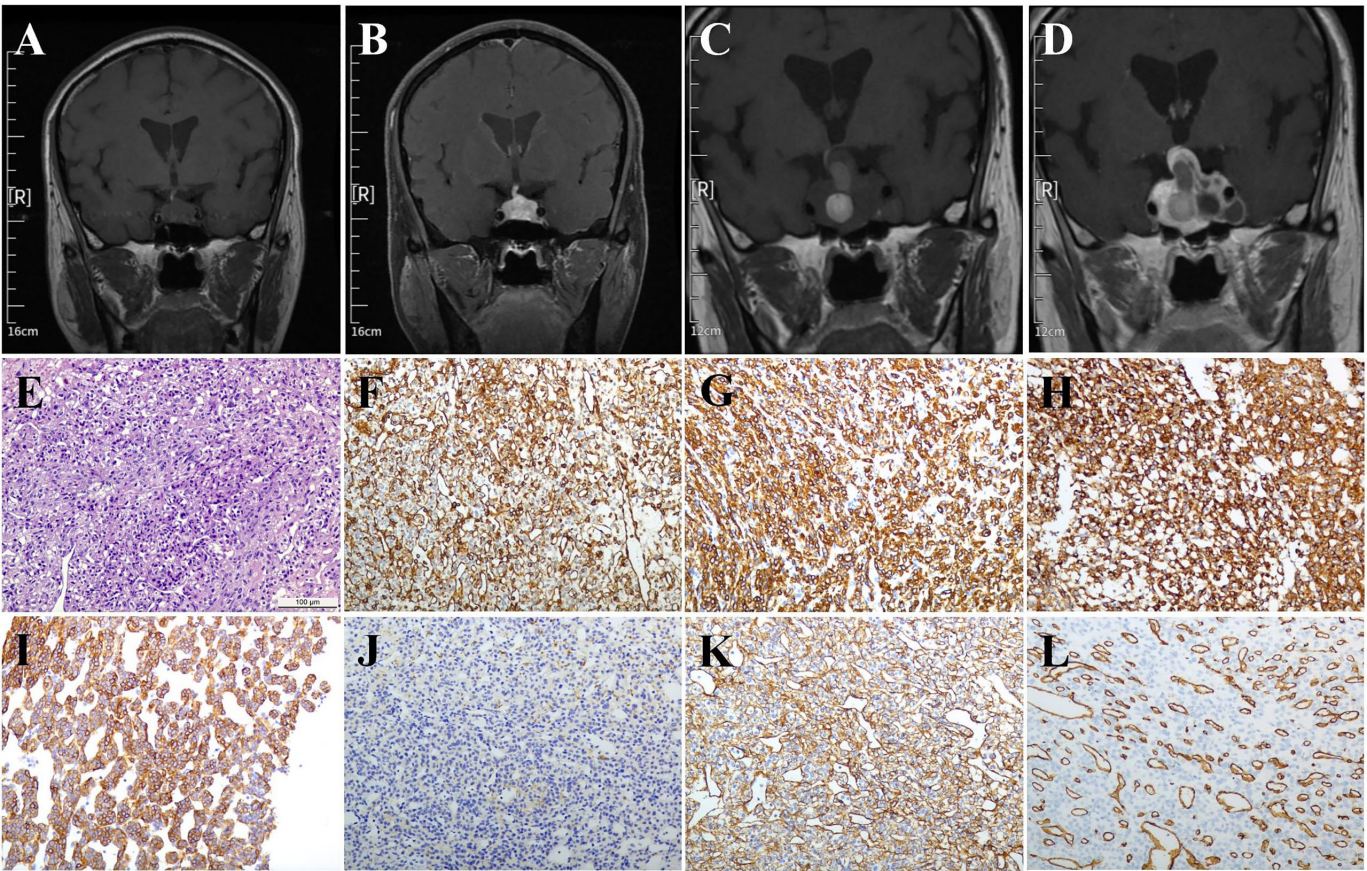
MRI and pathological images. **(A, B)** Five years before the operation: Coronal MRI and enhanced MRI images indicated the sellar lesions with a size of approximately 21.0 × 14.5 × 12 mm had cystic structures and was inhomogenously enhanced. **(C, D)** Three months before the operation: Coronal MRI and enhanced MRI images indicated the sellar lesions with a size of about 37 × 35 × 24 mm had variable signal intensity and multiple apoplectic events. **(E)** Hematoxylin-eosin (H&E) staining indicated the mild cell morphology with rare nuclear division; immunohistochemical staining indicated that the tumor cells were positive for **(F)** Vimentin, **(G)** SMA, **(H)** SYN, **(I)** h-caldesmon, **(J)** Desmin, **(K)** Collagen IV, and **(L)** CD34. Magnification, ×200; Bar = 100 μm.

A 69-year-old man presented with sellar mass for more than 5 years and visual deficits for about 3 months. Five years ago, the patient was hospitalized in the department of endocrinology due to hypopituitarism. During hospitalization, the high-resolution contrast enhanced MRI was performed and suggested an incidental lesion (21.0 × 14.5 × 12 mm) in the sellar region. The lesion had cystic structures and was heterogeneously enhanced, suggesting a “macroadenoma” (**Figures 1A, B**). However, the patient refused further surgery treatment. After discharge, the patient was followed up regularly clinically and radiologically. The sellar mass did not grow significantly, and no other symptoms appeared during follow-up. Three months ago, the patient presented with acute onset of diminished visual acuity and fields. The high-resolution contrast enhanced MRI revealed a giant hetereogenous sellar mass with multiple apoplectic events, measuring 37 × 35 × 24 mm in size (**Figures 1C, D**). In addition, the pituitary hormone test demonstrated low basal level of serum cortisol (8:00 AM, 0.54 μg/dl; normal, 6.7–22.6 μg/dl). On the basis of these findings, hypopituitarism secondary to a non-functional pituitary macroadenoma was suspected. Afterward, the patient underwent a neurosurgery *via* the endonasal endoscopic transsphenoidal approach (TSA) under general anesthesia. The visual acuity and field recovered soon after operation.

The paraffin sellar tumor specimen were cut (4 μm thickness), dewaxed, and then rehydrated. An antigen retrieval procedure was performed. Afterward, the sections were incubated in 3% H_2_O_2_ in phosphate-buffered saline (PBS) for about 10 min, blocked in PBS containing 5% normal goat serum at the room temperature for nearly 1 h, followed by incubation with the primary antibodies (**Supplementary Table 1**) at 4°C overnight. After washing three times, these sections were developed with the ABC kit and detected by Diaminobenzidine (DAB) staining (both from Vector Laboratories, Burlingame, CA, USA). Subsequently, the sections were stained with hematoxylin. Histopathological examination revealed mild cell morphology with rare nuclear division (**Figure 1E**) . Immunohistochemical (IHC) staining indicated tumor cells positive for Vimentin (**Figure 1F**), SMA (**Figure 1G**), SYN (**Figure 1H**), h-caldesmon (**Figure 1I**), Desmin (weak, **Figure 1J**), Collagen IV (**Figure 1K**), and CD34 (vascular, **Figure 1L**). Moreover, immunostains for AE1/AE3, Pit-1, ER, CgA, SF-1, T-pit, S-100, GFAP, EMA, STAT-6, and PAS were negative (data not shown).

## Discussion

Glomus tumor originates from the normal globular aberrant smooth muscle cells. It is a rare mesenchymal tumor with an organ-like structure (4). More than 96% of the tumor occurs in the fingertips, and mostly in the nail bed area. Glomus tumor was first reported by Wood in 1812. In 1924, Barre and Masson for the first time gave a relatively complete description of its histology, and put forward the term “glomus tumor”. In 1951, Kay et al. first reported a case of non-phalangeal glomus tumor, gastric glomus tumor (5). Since then, breast (6), penis (7), nerve (8), bone (9), lung (10), and other tissues glomus tumors have also been reported successively. In 1984, Asa et al. (11) first described the features of glomus tumors in the sellar region. Since this first description, other five cases have been reported successively (12–15), which are summarized in **Table 1**.

**Table 1:** Summary of the patients’ clinical data.

Year	Authors	Age	Sex	Symptom	Pituitary function	Treatment	Outcomes
1984	Asa et al. (11)	42	M	Decreased visual accuracy	Not Available	Surgery and radiotherapy	Recurrence
2005	Hanggi et al. (12)	47	F	Diplopia	Not Available	Surgery and radiotherapy	Recurrence
2011	Ebinu et al. (13)	72	M	Bitemporal hemianopia	Not Available	Surgery	
2020	Tsang et al. (14)	8	F	Decreased visual accuracy	Hypopituitarism	Surgery and Gamma Knife radiosurgery	Recurrence
2021	Quah et al. (15)	63	M	Blurred vision	Hypopituitarism	Surgery and radiotherapy	Not Available
		30	F	Intracranial hypertension	Hypopituitarism	Surgery	Death

Vascular spherules are composed of vascular cells, vascular structures, and smooth muscle tissues. According to the difference of composition proportions, glomus tumor is specifically subdivided into three subtypes: glomus tumor proper, glomangioma, and glomangiomyoma. According to the biological behavior, the tumor was classified as benign, uncertain malignant potential, and malignant. In the updated WHO classification, the criteria for malignant glomus tumor are as follows: (i) marked nuclear atypia and any level of mitotic activity; or (ii) atypical mitotic figures. Tumor should be categorized as uncertain malignant potential glomus tumor if it possesses any of the following characteristics: (i) tumor size >2 cm or deep location; (ii) atypical nuclear division (>5/50 HPF); (iii) atypical cells with round or fusiform appearance; and (iv) invading extra-capsular and surrounding tissues. In the current case, the tumor size was bigger than 2 cm, located in the sellar area, and had uncertain biological behavior, indicating that it was an uncertain malignant potential glomus tumor.

Because of the rare occurrence and non-specific clinical manifestations, the imaging may be still the most valuable method for the diagnosis of non-phalangeal glomus tumor. Considering that glomus tumor is filled with poorly circulated blood, MRI shows hypointensive signal on T1-weighted images, hyperintensive signal on T2-weighted images, and enhancement on T1-weighted images following gadolinium injection. As glomus tumor has a well-defined capsule, a more specific characteristic of a linear hyperintensive nidus surrounded by a rim of hypointensive signal could be showed on MRI. In a series of 42 glomus tumor patients study, MRI was reported to have a sensitivity of 90% and positive predictive value of 97% in diagnosis. However, the specificity was only 50%, and the negative predictive value was as low as 20% (16). In this case, the sellar mass showed clear tumor boundaries, obvious tumor capsule, mixed signals (mainly hypointensive signal) on T1-weighted images, mixed signals (mainly hyperintensive signal) on T2-weighted images, and heterogeneous enhancement with gadolinium on MRI scanning. Thus, the probable diagnosis of non-phalangeal glomus tumor should be considered, and further Digital subtraction angiography (DSA) examination is recommended.

The golden standard for treatment of glomus tumor is complete resection (17). According to the anatomical tumor position, we selected the transnasal TSA with endoscopic visualization. Unlike other solid sellar tumors, glomus tumor is a mesenchymal hemangioma essentially, which bleed easily during operation. Fortunately, the tumor was completely removed through concerted efforts of our multidisciplinary team (MDT), including experts from departments of anesthesiology, blood transfusion, and radiology. Imaging follow-up examination at 12 months after operation suggested that the tumor was resected totally without evidence of recurrence or metastasis.

## Conclusion

In conclusion, the current case reminds us that the glomus tumor should be considered as a differential diagnosis for sellar mass. Preoperative DSA examination can be performed if necessary. Surgical resection is the first choice for sellar glomus tumor. Notably, sufficient preoperative planning, including the image test, MDT discussion, hormone replacement therapy, and preoperative blood preparation, should be well prepared. Moreover, the long-term follow-up is needed due to the high recurrence rate, as evidenced in **Table 1**.

## Author Contributions

YC took charge of original draft writing. HT performed follow-up and management of the patient. ZBW contributed to manuscript review and editing. All authors contributed to the article and approved the submitted version.

## Conflict of Interest

The authors declare that the research was conducted in the absence of any commercial or financial relationships that could be construed as a potential conflict of interest.

## Publisher’s Note

All claims expressed in this article are solely those of the authors and do not necessarily represent those of their affiliated organizations, or those of the publisher, the editors and the reviewers. Any product that may be evaluated in this article, or claim that may be made by its manufacturer, is not guaranteed or endorsed by the publisher.
